# Anatomy

**Published:** 1879-04

**Authors:** 


*Collaborators:*

DR. H. GRADLE, DR. L. W. CASE, DR. R. PARK,
DR. R. TILLEY, DR. D. R. BROWER.

### ANATOMY.

ANATOMY OF THE MENINGEAL NERVES.—Duret. (*Brain.*, April, 1878.)

The following very lucid résumé of the origin and distribution of the meningeal nerves is extracted from a paper by Duret, "On the Rôle of the Dura Mater and its Nerves in Cerebral Traumatisms."

(*a.*) *Nerves of the Dura Mater.*—These all spring from the fifth pair. The *anterior* arise from the ethmoidal branch of the nasal and are distributed around the ethmoidal fossa and orbital roof. The *middle* spring directly from the Gasserian ganglion and proceed outwards, plunging immediately into the dura mater, nearer the internal than the external surface, ramifying in all directions over the temporo-sphenoidal region, and up to the parietal region of the membrane; several of the branches are lost in their course; two or three can be traced up to the vicinity of the superior longitudinal sinus. The *posterior* arise from the ophthalmic branch of Willis, not far from the Gasserian ganglion, cross the trochlear nerve, to which they are closely applied (some have thought they arose from this nerve), and arrive at the tentorium cerebelli, where they divide into external and internal branches.

The internal, two or three in number, course inwards so as at once to gain the base of the falx cerebri, into which they pass and ascend to gain the vertex. The external pass toward the lateral sinus and bend so as to reach the posterior part of the falx cerebri, in which they lose themselves.

Duret is inclined to the belief that the nervous filaments which are situated nearer the inner than the outer aspect of the dura mater end in special terminal organs, which increase the delicacy of their sensibility. The marked physiological troubles which ensue on their irritation lead to this belief.

(*b.*) *Nerves of the Arachnoid and Pia Mater.*—Luschka and Bochdalek have described nerve fibers in the arachnoid. According to Kölliker these filaments belong to the vessels of the pia mater. It is certain that a large number of the nerve fibres coming from the sympathetic accompany the carotid and vertebral arteries in the cranium.

Purkinje and Kölliker describe nerve plexuses at the base of the cranium surrounding the arteries of the circle of Willis. Their fibrils accompany the vessels which proceed from this, and are distributed with them over the whole of the pia mater. Kölliker claims to have followed them into the substance of the brain as far as vessels of ninety-nine, and less.

All these nerve fibers which accompany the arteries so far are the vaso-motor nerves of these vessels. They are the agents of reflex vascular disturbances so often occurring.

